# Mechanisms of action and synergetic formulas of plant-based natural compounds from traditional Chinese medicine for managing osteoporosis: a literature review

**DOI:** 10.3389/fmed.2023.1235081

**Published:** 2023-08-28

**Authors:** Chengcong Zhou, Shuchao Shen, Muxin Zhang, Huan Luo, Yuliang Zhang, Chengliang Wu, Lingfeng Zeng, Hongfeng Ruan

**Affiliations:** ^1^Institute of Orthopaedics and Traumatology, The First Affiliated Hospital of Zhejiang Chinese Medical University (Zhejiang Provincial Hospital of Traditional Chinese Medicine), Hangzhou, China; ^2^Department of Pharmacy, The Second Affiliated Hospital, Zhejiang University School of Medicine, Hangzhou, China; ^3^Hangzhou Fuyang Hospital of TCM Orthopedics and Traumatology, Hangzhou, China; ^4^Guangdong Provincial Hospital of Chinese Medicine, Guangzhou University of Chinese Medicine, Guangzhou, China

**Keywords:** osteoporosis, traditional Chinese medicine herbs, plant-based natural products, bone homeostasis, active ingredients, anti-osteoporosis drug

## Abstract

Osteoporosis (OP) is a systemic skeletal disease prevalent in older adults, characterized by substantial bone loss and deterioration of microstructure, resulting in heightened bone fragility and risk of fracture. Traditional Chinese Medicine (TCM) herbs have been widely employed in OP treatment owing to their advantages, such as good tolerance, low toxicity, high efficiency, and minimal adverse reactions. Increasing evidence also reveals that many plant-based compounds (or secondary metabolites) from these TCM formulas, such as resveratrol, naringin, and ginsenoside, have demonstrated beneficial effects in reducing the risk of OP. Nonetheless, the comprehensive roles of these natural products in OP have not been thoroughly clarified, impeding the development of synergistic formulas for optimal OP treatment. In this review, we sum up the pathological mechanisms of OP based on evidence from basic and clinical research; emphasis is placed on the *in vitro* and preclinical *in vivo* evidence-based anti-OP mechanisms of TCM formulas and their chemically active plant constituents, especially their effects on imbalanced bone homeostasis regulated by osteoblasts (responsible for bone formation), osteoclasts (responsible for bone resorption), bone marrow mesenchymal stem cells as well as bone microstructure, angiogenesis, and immune system. Furthermore, we prospectively discuss the combinatory ingredients from natural products from these TCM formulas. Our goal is to improve comprehension of the pharmacological mechanisms of TCM formulas and their chemically active constituents, which could inform the development of new strategies for managing OP.

## Introduction

Osteoporosis (OP) as a chronic systemic skeletal disease, is considered to be a growing silent epidemic in the 21st century, and it is estimated to affect approximately 200 million individuals worldwide (1, 2). It is characterized by a decline in bone mineral density (BMD) and degradation of bone tissue microstructure, which results in heightened bone fragility and a higher risk of fractures (3). Several factors, including hormonal imbalances, medication use (like glucocorticoids), smoking, lack of exercise, and insufficient calcium and vitamin D intake, contribute to bone loss in OP progression (4, 5). Epidemiology research has shown that 1/3 of women and 1/5 of men over the age of 50 are prone to osteoporotic fractures, with the risk increasing with age, particularly in women over 60 (6). Current OP treatments vary from non-pharmacological options (such as muscle tone and balance-improving exercises) to pharmacological therapies (such as bisphosphonates, denosumab, teriparatide, abaloparatide, and romosozumab) (7–9). Certain patients with low BMD or those who respond poorly to treatment may still have extremely low BMD despite receiving bone-formation therapy. The safety risks connected with these therapeutic agents have resulted in investigations into safer and more effective alternatives for treating OP.

Traditional Chinese medicine (TCM) has been used for over 2000 years to manage a broad spectrum of medical diseases or conditions and relies on plant-based natural products, including TCM formulas and their compounds and extracts (10–12). These products have been pivotal in maintaining the health of Chinese individuals, providing effective treatments for diseases and reducing adverse effects, compared to Western medicine or placebo, for instance, 5-Ling Granule produced significantly greater improvement in the severity of tic symptoms than placebo in an 8 weeks, double-blind, randomized, controlled trial and Pingchuan Yiqi granule significantly improves lung function and symptoms of acute asthma in a randomized, double-blind, placebo-controlled trial (13–17). As to OP, in clinical practice and animal experiments, TCM formulas, such as Bushen Huoxue decoction (BSHXD), Er Xian Decoction (EXD), and Liuwei Dihuang Pill (LWDHP), have been proven to be effective in treating OP (18–20).

Meanwhile, various natural compounds, which are present in TCM formulas, have exhibited diverse biological effects in treating osteoarticular degenerative diseases, including OP. Many of these compounds have been proven to have effects akin to those of the original formulas for treating OP (21–26). Of note, chemically active ingredients from plant-based compounds (or secondary metabolites), like resveratrol, naringin, and ginsenoside, have been extensively employed for preventing and treating OP, as corroborated by their beneficial effects in decreasing bone resorption, increasing bone formation, repairing bone microstructure, enhancing angiogenesis, and improving the immune system (27–29). Nevertheless, the full extent of the roles played by these TCM herb formulas and their natural products in OP remains to be systematically elucidated, which hampers the development of synergistic formulas for more effective OP treatment.

Based on evidence from clinical and basic research, this review outlines the underlying pathological mechanisms of OP, and offers a concise summary of the most recent discoveries on the anti-OP mechanism of TCM formulas and their chemically active ingredients, highlighting their detailed effects on osteoblasts and osteoclasts, bone marrow mesenchymal stem cells and bone microstructure, angiogenesis, and the immune system. Furthermore, through the prospective discussion of the unique ingredient combinations in these TCM formulas, we seek to offer valuable information for devising fresh OP treatment strategies.

## Mechanism of OP

**Figure 1:**
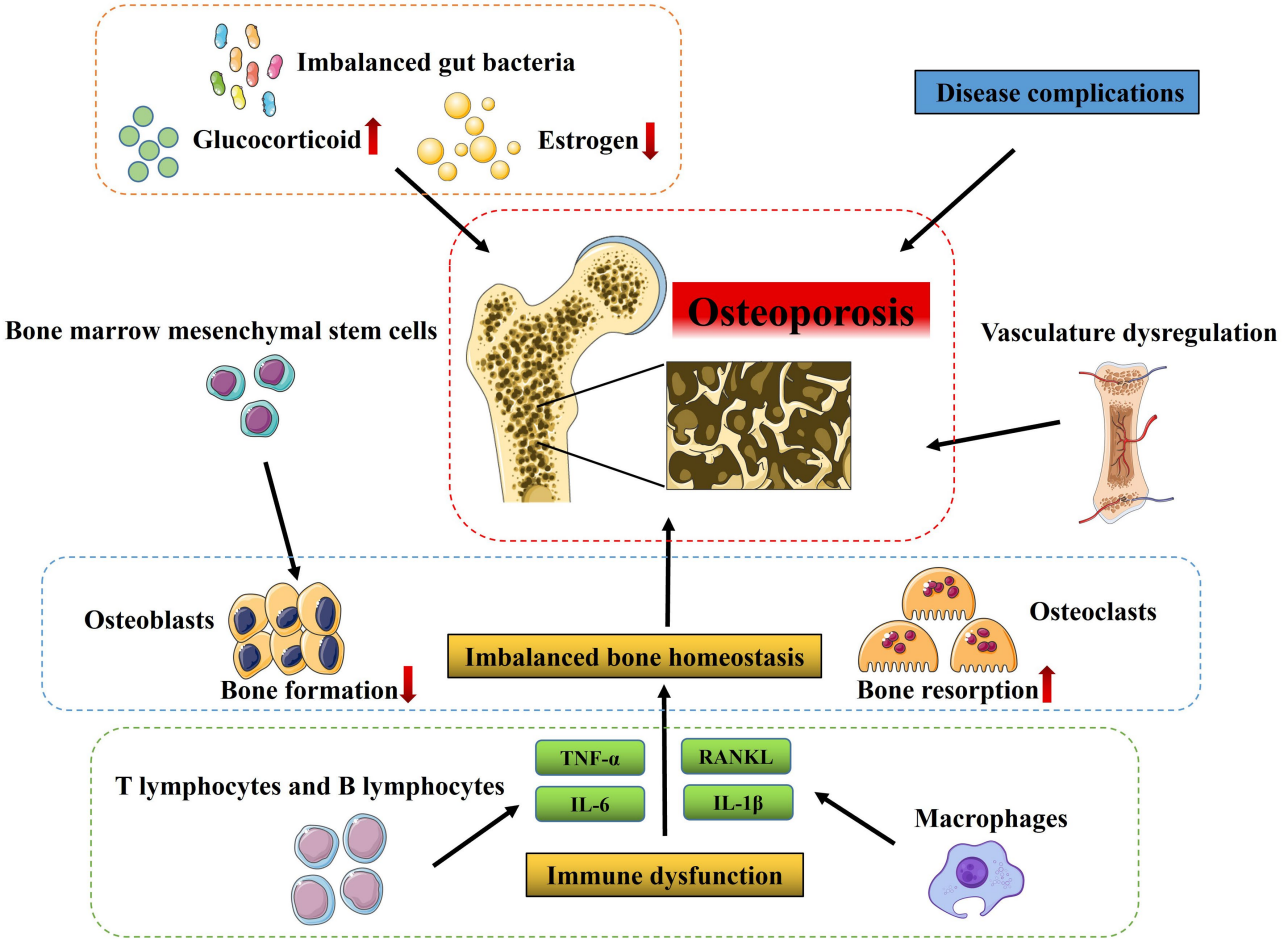
Pathological mechanisms of osteoporosis. Various factors contribute to OP progression, including imbalanced bone homeostasis regulated by osteoblasts (responsible for bone formation), osteoclasts (responsible for bone resorption), bone marrow mesenchymal stem cells as well as vasculature dysregulation, and immune dysfunction and many other factors such as estrogen deficiency, long-term and high-dose glucocorticoid treatment and imbalanced gut bacteria.

OP is diagnosed when an individual’s BMD T score falls at or below −2.5, according to WHO diagnostic criteria (30). Mechanistically, various factors contribute to OP progression, including bone and hormone metabolism disorders, modulation of signaling pathways, dysregulation of the vasculature, immune dysfunction, and other factors that can vary (31–33). Here, we mainly summarize the potential pathological mechanisms of OP from various aspects mentioned above (Figure 1).

### Bone homeostasis

The balance of bone-forming and bone-resorbing activities is essential to preserve bone homeostasis. Multiple bone cell types, including osteoblasts (OBs), osteoclasts (OCs), and bone marrow mesenchymal stem cells (BMSCs) are involved in the multifaceted process of bone homeostasis (34, 35). OBs and BMSCs are responsible for bone formation, whereas OCs are the primary functional cells involved in bone resorption (36). This interdependent mechanism is referred to as coupling, where OCs break down the organic matrix and dissolve bone minerals, followed by the recruitment of OBs to deposit fresh bone matrix that later mineralizes. An imbalance in the bone remodeling process, leading to excessive bone resorption relative to bone formation, is the principal factor contributing to the greater occurrence of OP (37, 38). The normal bone remodeling equilibrium can be disrupted by various factors, such as hormones, endocrine regulation, cytokines, and others, which interfere with OBs and OCs differentiation and activity (39, 40). Furthermore, the imbalance in bone remodeling also deteriorates the bone microstructure, making it a target for therapeutic approaches to improve bone strength and decrease the risk of fractures (41).

Besides, the regulation of OP initiation and development involves various signaling pathways, incorporating genetic elements, and modulatory molecules, like TGF-β, RANKL, as well as BMP, which have been studied in great detail over the past few decades (42). These signal transduction pathways are implicated in regulating the differentiation and growth of OBs, OCs, or BMSCs. For example, the Wnt/β-catenin signaling pathway plays a crucial role in the differentiation of BMSCs into OBs by determining their directionality, whereas knocking down *β-catenin* expression significantly elevates OCs numbers and accelerates bone resorption rates, resulting in the development of OP (42, 43). In addition, TGF-β signaling not only inhibits OBs maturation, mineralization, and transition into osteocytes, but also inhibits OCs differentiation by decreasing RANKL/OPG secretion ratio (44, 45). Other signaling pathways, including Notch, and BMP2/SMADs, also participate in bone homeostasis and are highly correlated to the differentiation, growth, and maturation of OBs, OCs, and BMSCs (46–48). In general, dysregulation of signaling pathways mentioned above is associated with the risk of OP.

### Vasculature

The existence of blood vessels in the bone microenvironment is necessary for appropriate bone development and growth, post-fracture healing, and maintaining healthy bones. Angiogenesis in bone tissues involves the proliferation, migration, and tube formation of endothelial cells, which establishes blood flow conduits that deliver essential nourishment, vital oxygen, crucial growth-promoting substances, and hormones to bone cells (31). On the contrary, disruption in angiogenesis in bone tissues can disturb the equilibrium within the bone niche, which is intimately linked to the pathological progression of OP (49, 50). Emerging clinical evidence reveals that individuals with OP have comparatively decreased blood supply to the bone than those with normal bone density, suggesting a significant correlation between bone mineral density and bone vascularization (51). Thus, a significant hallmark of OP is a reduction in the number of sinusoidal and arterial capillaries located in the bone marrow, resulting in decreased delivery of blood to the bone tissue (52). Moreover, the reduction in blood perfusion within the bone marrow is also linked to a decline in BMD and an increased incidence of fractures in elderly women (53). Similarly, age and ovariectomized (OVX) mice show decreased levels of PDGF-B in serum and bone marrow, along with a marked decrease of type H vessels in long bones (54, 55). All these findings indicate a cause-and-effect connection between the number of type H vessels and OP, given the type H vessels’ capability to promote angiogenesis and bone formation, and maintain the delicate equilibrium between bone resorption and formation (54, 56). Therefore, activating angiogenesis might be a vital therapy strategy in the prevention of OP.

### Immune system

Bone surrounds the bone marrow, which serves as an essential center for the immune system’s maturation and hematopoiesis. It is well-documented that there is a significant connection between the immune system and the skeletal system. T cells and B cells are the two types of lymphocytes responsible for adaptive immunity. They and their cytokines can impact the functions and activities of both OBs and OCs, with additional indirect implications on OCs due to an increase in RANKL expression via OBs proliferation (57–59). Moreover, these two systems share several regulatory molecules, including cytokines and signaling molecules. The innate immune cells are major producers of pro-inflammatory mediators, with several noteworthy cytokines such as IL-6, TNF-α, IL-1β, as well as ROS that have a crucial impact on OP (60). For instance, TNF-α can promote OCs formation by directly interacting with both OCs along with their precursors, synergistically with RANKL (61). Noteworthy, the regulation of bone homeostasis involves the contribution of multiple immune factors, such as T cells, B cells, NK cells, macrophages, and many other cytokines (37, 62). Different subsets of T cells, namely CD4^+^ T cells and CD8^+^ T cells, all exert crucial impacts in regulating bone health. Of these, Th17 cells stand out as a significant cause of bone loss since they produce significantly more RANKL and TNF-α (63, 64). Besides, the presence of B cells and their production of RANKL could be involved in the pathogenesis of ovariectomy-induced bone loss (65). Likewise, M1 macrophages exert a significant impact on promoting osteoclastogenesis by producing abundant ROS and pro-osteoclastogenic cytokines such as TNF-α and IL-1β (66). In contrast, unlike M1 macrophages which promote osteoclastogenesis, M2 macrophages contribute to bone mineralization by promoting the differentiation of MSCs and precursor OBs into mature OBs, thus supporting bone health (67).

### Other factors

Accumulating evidence reveals that the development of OP is also affected by various other factors, such as disease complications, estrogen levels, and gut microbiota (68). Among them, endocrine systemic diseases, hematological diseases, or other metabolic diseases, have been shown to be closely related to the occurrence and development of OP (68). It has been reported that diabetic patients may suffer reduced bone turnover and bone loss due to deleterious impacts on bone metabolism and degradation of the extracellular matrix. And the development of brittle bone in diabetes is controlled by many contributing elements, such as obesity, insulin resistance, dysglycemia, myopathy, and the use of certain medications. In parallel, certain comorbidities, including thyroid dysfunction, gonadal disorders, and maldigestion, may disrupt bone metabolism and contribute to an imbalance in affected individuals (69, 70). Given the close relationships between bone and bone marrow, hematological diseases exert a significant influence on the secondary forms of OP; there is an increased rate of bone resorption among patients with hemophilia (71). Additionally, estrogen is considered to act directly on bone cells in preventing a decline of bone mass (72, 73), and it directly or indirectly influences immune cells to impact OBs and OCs via additional intermediaries like OPG/RANKL and chemokines such as TNF-α and IL-1β (74). Moreover, growing evidence indicates there is a connection between a balanced microbiome and stable bone health, and that an imbalance in gut bacteria might worsen the function of OCs, thus resulting in OP (68).

Furthermore, clinical evidence reveals that long-term and high-dose glucocorticoid treatment is another significant contributor to OP (75). Accumulating studies have demonstrated that glucocorticoids can inhibit OBs proliferation and increase rates of apoptosis of OBs, inhibit calcium uptake in the gastrointestinal tract, act as an antagonist to vitamin D, upregulate RANK ligand, and suppress OPG (76, 77). Importantly, glucocorticoid therapy also has been shown to adversely affect muscle mass and muscle strength, increasing the risk for OP fractures (78, 79).

## Anti-OP effects of TCM and plant-based natural compounds

Compared with chemically synthesized drugs, TCM possesses beneficial characteristics such as fewer adverse reactions, long-term use, and stable treatment effect, therefore, TCM is increased in clinical trials to treat OP, among them, Bushen Zhuanggu tablet was demonstrated to be effective in the postmenopausal OP treatment by increasing the BMD, as well as regulating calcium and phosphorus metabolism in case-control studies (80–84). TCM formulas, including LWDHP, BSHXD, and other classic formulas, have demonstrated significant clinical effectiveness in both preventing and treating OP (20, 85–88). Given the multifaceted and diverse pathological mechanisms of OP, comprehensively understanding the pharmacological mechanisms of diverse TCM approaches for OP treatment is imperative. Here, we summarize *in vitro* and preclinical *in vivo* evidence-based anti-OP mechanisms of TCM formulas and their chemically active ingredients from various perspectives such as OBs and OCs, BMSCs, as well as bone microstructure, angiogenesis, and immune system regulation.

### The anti-OP effects of TCM formulas

In general, many TCM formulas have the effect of improving OP phenotypes to some extent by promoting bone homeostasis, and angiogenesis, and regulating the immune system (Table 1). In terms of TCM formulas for the treatment of OP, LWDHP increases the BMD of femurs and improves the biomechanical capability of the vertebral body in OVX rats, mechanically, it could upregulate Runx2 and Osx expression and augment the number of calcified nodules in OBs by activating canonical Wnt/β-catenin cascade through the increase of Lrp-5 and β-catenin expressions (89). Bioinformatics analysis also provides potential therapeutic targets of LWDHP against OP such as AKT1, ATF2, and FBXW7 from a systemic perspective (90, 96). Qing’e Pill (QEP) inhibits OB ferroptosis and increases the transcription of osteogenesis-related genes, such as *BMP2*, *Runx2* to increase the number of trabecula and trabecular connections by activating PI3K/AKT pathway and inhibiting ATM Serine/Threonine Kinase expression, thereby exerting a therapeutic role in OVX rats (97). In parallel, *in vitro* studies of OBs cultured with Danggui Buxue Decoction (DGBXD) led to a significant improvement in mitochondrial function, antioxidation, and anti-inflammatory, leading to increased OBs activity (91, 92).

**Table 1:** The efficacy of TCM formulas for treating OP *in vivo*.

TCM formula	Model	Dosage and duration	Results	References
LWDHP	OVX	2 mL/100 g/day; 12 weeks	Increases expression of Lrp-5, β-catenin, Runx2, Osx and the BMD of femurs	(89)
QEP	OVX	4.5 g/kg/day; 2 weeks	Promotes the osteogenic protein expression and calcium-phosphate metabolism and inhibits osteoclast function	(90)
BSHXD	GIOP mice	16 mg/kg, every 2 days; 6 weeks	Inhibits osteoclast formation and function by suppressing ERK, JNK, and NF-κB signaling	(91)
JQBD	IBD-induced bone loss model	16.5 g/kg/day; 2 weeks	Inhibits activation of the RANK/RANKL/OPG signaling pathway	(92)
YGY	OVX	20 mL/kg/day; 12 weeks	Inhibits bone loss and osteoclastogenesis, decreased the expression of RNAKL and NF-κB signaling genes	(93)
ZGP	GIOP rats	1.62 g/kg/day; 3 months	Inhibits bone resorption and accelerated bone formation through the activation of let-7f and regulation of autophagy-associated genes	(94)
EXD	OVX	300 mg/kg/day; 12 weeks	Ameliorates OVX-induced bone loss and bone microstructure deterioration, upregulates the level of serum estrogen	(95)
BSTLD	OVX	6 g and 12 g/kg/day; 12 weeks	Reduces osteoclasts activation and bone resorption, reduced mRNA and protein levels of calcitonin receptor and CTSK	(53)

As another part of bone homeostasis, OCs are also extensively studied in treated with TCM formulas. TCM formulas, BSHXD, and Jianpi Qingchang Bushen decoction (JQBD) can alleviate bone loss, decrease bone volume, and limit osteoclastogenesis by reducing the RANKL-stimulated NF-κB, ERK, JNK signal transduction pathways in OP model mice (93, 98). Bushen Tongluo decoction (BSTLD) and You Gui Yin (YGY) can promote bone generation and attenuate OC activity and bone resorption in the OVX-induced OP mouse model by reducing RANKL/OPG ratio (53, 99).

Meanwhile, numerous studies have demonstrated that TCM formulas can promote the BMSCs’ growth and migration to alleviate OP symptoms by enhancing the ability of BMSCs to differentiate into OBs. Duhuo Jisheng Decoction (DHJSD) was found to promote the activity of BMSCs, leading to upregulation of the expression of *BMP2* and *Runx2* genes, and OBs differentiation through activating SMAD1/5/8 and ERK signaling pathway (94). Taohong Siwu Decoction (THSWD) enhances the proliferation, and migration, together with bone-forming potentiality by upregulating VEGF expression and the phosphorylation of FAK and Src (100). Zuogui Pill (ZGP), another classical TCM prescription, could up-regulate *let-7f* and *Runx2* mRNA expression and down-regulate *Beclin-1*, *ATG12*, *ATG5*, *LC3*, and *CTSK* mRNA expression in BMSCs to promote osteogenic differentiation of BMSCs, thus improving the OP progression (95), while another study indicated it can also slow down the senescence of BMSCs through modulating Wnt/β-catenin signaling pathway (101).

Moreover, EXD, which has been extensively used for the treatment of OP (102), can improve the bone microstructure and deterioration of bone loss in the OVX-induced OP model through lipid metabolism and the IGF1/PI3K/AKT pathway (103). BSTLD treatment results in a significant promotion in angiogenesis in OVX-induced OP rats, which could be associated with the promotion of HIF-1α/VEGF-regulated angiogenesis signal transduction and inhibition of the RANKL/OPG ratio (53). In a clinical trial, LWDHP has been found to promote the expression of immune-related cardiotrophin-like cytokine factor 1 (CLCF1) in the peripheral blood of postmenopausal osteoporosis patients with kidney Yin deficiency by activating the JAK/STAT signaling pathway, thereby improving immunocompetence, which provides a promising therapeutic approach for postmenopausal OP (104). Other classic Chinese herbal medicine like Xianling Gubao Capsule, Zhuanggu Zhitong Capsule, and Guilu Erxian Glue were also demonstrated to provide potential therapeutic benefits for the treatment of OP through *in vivo* and *in vitro* research (105–108).

### The anti-OP effects of herbs and chemical ingredients in TCM formulas

Extensive *in vivo* studies have demonstrated the anti-OP effects of various Chinese herbs and their extracts, including *Rhizoma Drynariae* homogenous polysaccharide, mixed extracts of *Fructus Corni* and *Radix Achyranthis Bidentatae* (the main components of LWDHP and ZGP), *Rhizoma Drynariae*. These herbs have been shown to ameliorate the OP phenotype by increasing BMD and bone mineral content, which is closely related to the activity of OBs (109–111). Resveratrol, derived from *Reynoutria japonica*, has been found to exhibit significant antioxidant capacity in OBs by triggering the NRF2/SIRT1/FoxO1 signaling pathway and improving excessive iron-induced oxidative stress (112–114). Puerarin, another bioactive ingredient extracted from the root of *Pueraria lobata (Willd.) Ohwi*, has been found to significantly upregulate the expression of *ALP* and *OPG* mRNA levels while inhibiting OBs apoptosis (115, 116). Similarly, Icariin, a compound rich in TCM formulas like EXD, can also promote OBs proliferation, differentiation, and mineralization, as demonstrated by increased expression of ALP and Col I, and bone nodule formation but inhibit their apoptosis by activating ERK and JNK signaling but not p38 signaling (117). Intriguingly, in another study, MC3T3-E1 subclone 14 cell line administered with Icaritin, a hydrolytic product of Icariin, increased mRNA levels and protein expression of ALP, COL1, OC, OPN, and RUNX2 to enhance preosteoblastic cell differentiation, and upregulated the bone nodule formation and collagen synthesis to improve mineralization through the activation of ERK and p38 signalings but not the JNK signaling (118). These discrepancies in reported signaling activation can be attributed to several factors, including differences in the compounds used, varying concentrations, and inconsistent timing of signal detection between the studies (119).

OCs and a major osteoclastogenic molecule, RANKL, have also been studied in relation to the effective monomer and active ingredients from TCM formulas. For instance, it has been suggested that dendrobium, a traditional medicinal plant, exhibits potent suppression of OCs by inhibiting the increase in ROS, the expression of c-fos, and NFATC1, which are mediated through RANKL, and significantly blocking *Mmp9* RNA transcription to reduce LPS-stimulated inflammatory bone loss (120). Moreover, Schisandrin A, which is rich in BSTLD, can inhibit the advantage of ROS induced by RANKL on OCs to improve bone resorption in OVX-induced OP mice via stimulating Nrf2 activity (121). Ellagic acid inhibits the expression of genes and proteins exclusive to OCs and hinders OCs differentiation, which limits bone loss, by blocking RANKL-RANK ligation, along with RANKL-conducted osteoclastogenesis (122, 123). In parallel, Aconitine, Berberine, and dihydro-artemisinin can inhibit RANKL-mediated osteoclastogenesis and bone resorption activity, leading to the promotion of osteogenic recovery (124–126).

Moreover, some TCM has been suggested to have dual effects. For example, Icariin can alleviate mitochondrial membrane potential dysfunction and oxygen-free radical production, promote Runx2, ALP, and OPN expression in OBs caused by excessive iron accumulation, and additionally inhibit the cellular differentiation and activity of OCs (127).

As for BMSCs, Artemisinin exerts antioxidant effects on BMSCs by inhibiting hydrogen peroxide-induced Caspase 3 activation and apoptosis of BMSCs (128). Likewise, Leonurine attenuates oxidative stress-induced COX2 and NOX4 mRNA expression in BMSCs and up-regulates mitochondrial membrane potential levels through the PI3K/Akt/mTOR signaling cascade to enhance the growth and differentiation capability of rat BMSCs (129, 130). Salvianolic acid B, extracted from *Salvia miltiorrhiza*, has been investigated in human BMSCs that can increase the expression of ALP, OPN, and Runx2 while promoting the formation of bone minerals (131). In addition, Panax notoginseng saponins dose-dependently increased ALP activity and ALP, *Cbfa1*, *OC*, and *BSP* mRNA levels of BMSCs, and decreased the mRNA and protein expression of PPARγ2, so as to accelerate proliferation and osteogenic differentiation of BMSCs (132). Meanwhile, it can also downregulate the number of OC precursor cells to inhibit bone resorption, together with enhancing BMSCs differentiation activity in the direction of osteogenesis (133). Oral administration of Ginkgolide has been identified as increasing the OPG-to-RANKL ratio and thus improving bone mass in three animal OP models, including the aging-, OVX-, and glucocorticoid-induced OP models (134, 135).

The destruction of bone microstructure is one of the typical characteristics of OP, and thus, the repair of bone microstructure is a standard to measure medicinal effectiveness. α-asarone, which is extracted from a TCM herb *Acorus tatarinowii*, is capable of inhibiting osteoclastogenesis and strengthening bone microstructure in OP models induced by estrogen deficiency (136). Cuscuta extract, which is from TCM formulas such as EXD and BSHXD, indicates an anti-OP effect by increasing bone density and bone microstructure via the c-fos/c-Src kinase/NFATC1 signaling pathway in OVX-induced OP mice (137). As mentioned above, the Wnt/β-catenin signaling pathway exerts a key role in the development of OP. Studies have shown that Ginsenoside Rc and Rhizoma drynariae total flavonoids noticeably hinder the decline of bone mass and bone volume, and promote osteogenesis and bone formation via the Wnt/β-catenin signaling pathway (138, 139). Moreover, Bionic Tiger-Bone Powder promotes osteogenesis, inhibits OCs, and increases collagen content in OVX-induced OP mice, improving bone microstructure and biomechanical strength. In line with the *in vivo* study, it also increases BMD in postmenopausal patients with OP in clinical (140). In different animal OP models, current evidence also suggests that TCM has preventive effects on OP by improving bone microstructure. Ganoderma lucidum contained in EXD can prevent extra bone loss and improve bone microstructure in OVX-induced OP mice by regulating bone and adipose tissue homeostasis, which can prevent bone loss caused by estrogen deficiency (141). Radix Achyranthis Bidentatae with Eucommiae Cortex herbal pair ameliorates glucocorticoid-induced OP by modulating the expressions of Runx2, OP-1, and β-catenin (142). In terms of the disuse-induced OP model, Scutellaria extract can significantly improve bone density and mechanical strength to prevent OP in the hindlimb unloading tail-suspended rat model (143). And oral administration of total flavonoid extracts from epimedium increase maximum BMD in normal-growing rats (144).

There is increasing evidence that angiogenesis is intimately associated with bone generation and remodeling to ensure adequate bone homeostasis. Thus, the promotion of angiogenesis-dependent osteogenesis and the reduction of bone loss are essential for preventing and managing OP (54). Studies have shown that Naringin derived from the traditional Chinese medicinal plant *Drynaria* regulated the function of endothelial cells and promoted angiogenesis, thereby exhibiting an anti-osteoporotic effect in postmenopausal osteoporotic rat models (145). As an active component found in many TCM herbs, vitexin modulates both osteogenesis and angiogenesis in an OVX-induced OP rat model through vitamin D receptor and endothelial NO synthase pathway (146). It is well-recognized that diabetes mellitus raises the risk of OP, and TCM also contributes to treating diabetic OP via angiogenesis. Curcumin prevents bone loss and promotes angiogenesis in diabetic OP mice by suppressing the hyper glucose-activated NF-κB pathway to restore osteogenic and angiogenic coupling of BMSC in hyperglycemia (147). In addition, Ginsenoside Rg1 facilitates the secretion of VEGF, triggers the Noggin/Notch signaling pathway, and enhances the interaction between H-type blood vessels and bone formation, leading to an improvement in bone structure in a mouse model of diabetic OP (148). Besides, Salvianolic acid B and Salidroside were also reported to prevent bone loss by enhancing angiogenesis in Glucocorticoid-induced and OVX-induced OP model, respectively, (149, 150).

The term “osteoimmunology” was coined in a commentary in *Nature* to highlight the connection point between osteology and immunology (151). In recent decades, mounting evidence suggests that TCM herbs and their extracts can regulate the immune system, which is essential for effective OP treatment. Ganoderma lucidum mycelium extract Ling-Zhi-8, an immunomodulatory protein, can effectively alleviate glucocorticoids-induced OP phenotypes in mice, as indicated by the increase in the OPG/RANKL ratio, suppression of osteoclastogenesis, and improvement in serum mineral metabolism, bone formation, and absorption markers expression, as well as expressions of hormone molecules such as estradiol and parathyroid hormone (152). Osthole, a type of coumarin compound present in various medicinal plants, including *Cnidium monnieri*, can enhance the immunosuppressive capacity of osteoporotic BMSCs to improve the therapeutic effect of BMSCs transplantation on colitis as well as OP (153). Likewise, *Cissus quadrangularis* markedly promotes the production of immune cells that counteract osteoclastogenesis, including Th1, Th2, and Tregs, in the bone marrow, while simultaneously decreasing the number of OC-promoting Th17 cells. This effectively inhibits RANKL’s ability to generate OCs and hinders the capacity of OCs in bone resorption (154).

Given the importance of proinflammatory cytokines, including TNF-α, IL-1β, as well as IL-17 in osteoimmunology, there is accruing evidence from recent studies suggesting that TCM herbs and their active ingredients such as Sinomenine (155), and Galanin (156) have therapeutical effects on OP by regulating the inflammatory reaction and inflammation-related signaling pathways. We have summarized the chemically active constituents in TCM herbs for treating OP in Table 2.

**Table 2:** The chemically active constituents in TCM herbs for the therapy of OP.

Active constituents	Medicinal materials	Antiosteoporotic action	References
Resveratrol	*Reynoutria japonica*	Exhibits antioxidant capacity in OBs and improves excessive iron-induced oxidative	(105–107)
Puerarin	*Pueraria lobata (Willd.) Ohwi*	Increases the expression of *ALP* and *OPG* mRNA levels and inhibits OBs apoptosis	(108, 109)
Icariin	*Herba epimedii*	Inhibits the apoptosis of OBs by activating ERK and JNK signaling	(110)
Schisandrin A	*Schisandrae chinensis* Fructus	Improves bone loss in OVX mice by stimulating the activity of Nrf2	(114)
Aconitine	*Aconitum*	Inhibits RANKL-induced OCs formation and bone resorption activity	(118)
Artemisinin	*Artemisia annua* (Asteraceae)	Exerts antioxidant effects on BMSCs and inhibits hydrogen peroxide-induced apoptosis of BMSCs	(121)
Leonurine	*Herb leonuri*	Up-regulates mitochondrial membrane potential levels and improves the proliferation and differentiation ability of rat BMSCs	(122, 123)
Salvianolic acid B	*Salvia miltiorrhiza*	Increases the expression of ALP, OPN, Runx2, and Osx, and promotes osteogenic mineralization	(124)
Panax notoginseng saponins	*Panax notoginseng*	Increases ALP activity, downregulates the number of OC precursor cells to inhibit bone resorption, and promotes the differentiation of BMSCs	(125, 126)
Ginkgolide B	*Ginkgo biloba*	Increases the ratio of OPG-to-RANKL and improves the bone mass	(127, 128)
α-asarone	*Acorus tatarinowii*	Inhibits OCs generation and improves the bone microstructure	(129)
Ginsenoside Rc	*Panax ginseng*	Increases bone density and bone volume fraction, and promotes bone formation and osteogenic differentiation through the Wnt/β-catenin signaling pathway	(131)
Naringin	*Drynaria*	Regulates the function of endothelial cells and promotes angiogenesis	(138)
Vitexin	*Crataegus pinnatifida* (hawthorn)	Regulates angiogenesis and osteogenesis in ovariectomy-induced OP rats through VDR/eNOS signaling pathway	(139)
Curcumin	*Curcuma longa* L	Prevents bone loss and promotes angiogenesis by inhibiting the NF-κB signaling pathway	(140)
Ginsenoside Rg1	*Panax ginseng*	Promotes VEGF secretion, activates Noggin/Notch pathway and improves bone structure	(141)
Ling-Zhi-8	*Ganoderma lucidum*	Alleviates the symptoms of glucocorticoids-induced OP, and increase the OPG/RANKL ratio to limit the production of OCs	(145)
Osthole	*Cnidium monnieri*	Enhances the immunosuppressive capacity of osteoporotic BMSCs to improve the therapeutic effect of BMSCs transplantation	(146)

## Conclusion and perspectives

**Figure 2:**
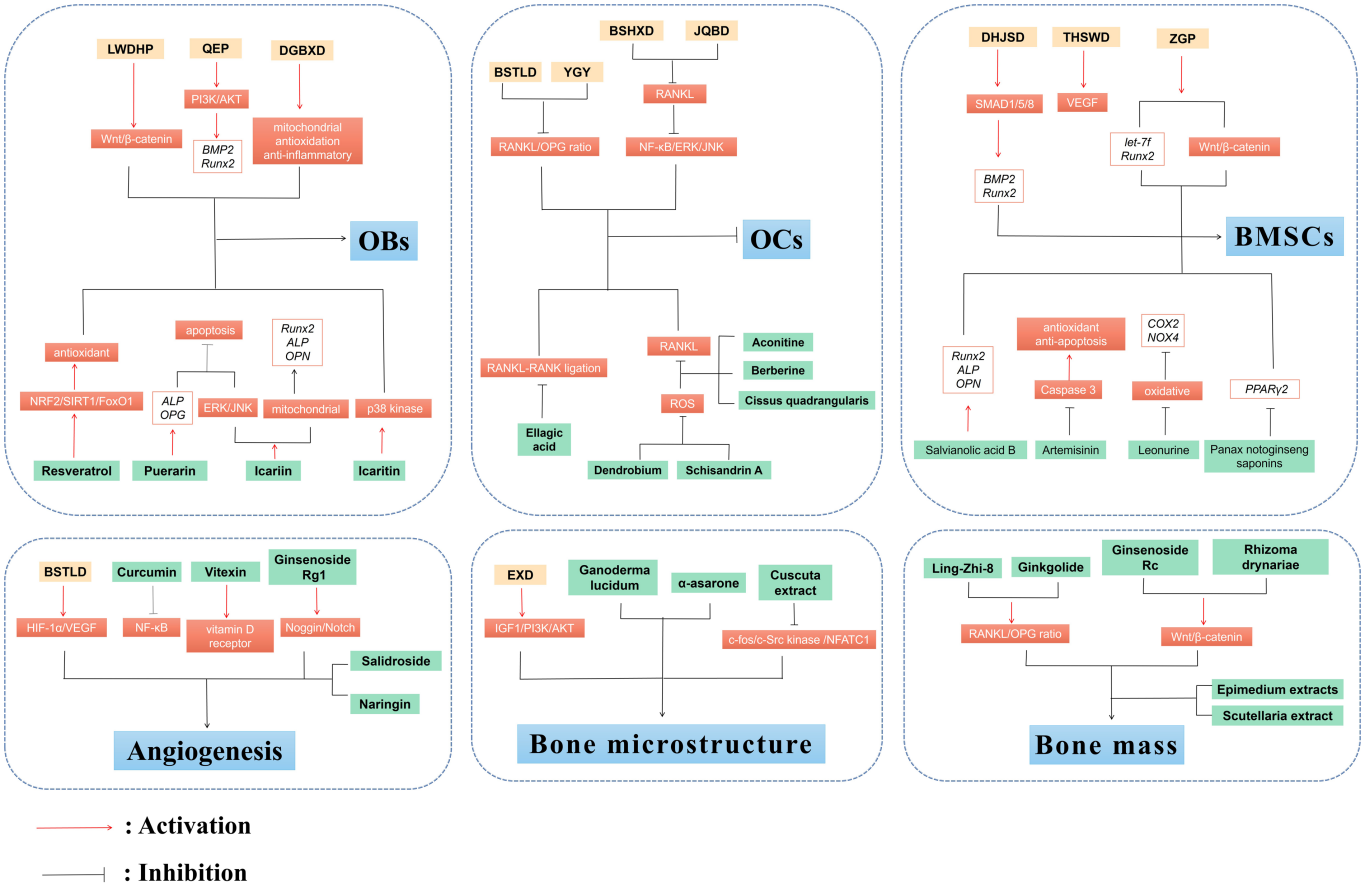
Anti-OP effects of TCM and plant-based natural compounds. TCM and plant-based natural compounds play a therapeutic role on OP from various perspectives such as OBs and OCs, BMSCs, bone microstructure, angiogenesis and bone mass.

The main feature of OP is the pathological decrease of BMD caused by the imbalance in bone remodeling, which involves a sophisticated interplay of endogenous, exogenous, mechanical, biological, and immunological mediators. TCM formulas are rich in natural compounds and display diverse biological effects in treating various diseases. Importantly, clinical trials as well as *in vivo* and *in vitro* findings demonstrate that TCM formulas such as LWDHP, QEP, EXD, YGY, and their chemically active constituents such as Resveratrol, Puerarin, Icariin, Schisandrin A possess the potential to deliver beneficial outcomes in the management of OP. Mechanically, they primarily reverse the OP progression by regulating bone remodeling, bone homeostasis, and angiogenesis, as well as OP-related signaling pathways and immune factors (Figure 2).

Compared to chemical drugs, TCM formulas and their active ingredients possess the advantages of producing fewer adverse effects and being appropriate for sustained usage. TCM formulas containing multiple herbs, in which each single herb medicine often contains anti-OP constituents, have the potential to advance our understanding of traditional medicine’s role in modern healthcare and accelerate the screening of new anti-OP drugs. Also, the combination of chemical drugs and TCM formulas might contribute to discovering effective therapies for treating OP.

It should be noted that treating OP requires a comprehensive understanding of its etiology. Thus, various animal models of OP, including OVX-induced OP models, chemically-induced OP models, and disuse-induced OP models, are frequently utilized to study the underlying mechanisms of this bone disorder. TCM formulas and their chemically active ingredients should be tested in multiple models to comprehensively elucidate their anti-OP effects. Although estrogen substitution therapy can prevent OP, the anti-OP mechanisms of many estrogen-like TCM formulas (such as Shugan Liangxue decoction, Qibao Meiran formula) or monomers (such as *Radix astragali*, *Curcuma comosa Roxb*) are still unknown (157–160). Further studies elucidating the anti-OP mechanism of these formulas or monomers targeting estrogen-like effects will greatly promote the treatment of OP with TCM. Moreover, investigating the effects of TCM on the factors that regulate estrogens and their receptors will aid in screening new anti-OP drugs. In the past few years, non-coding RNAs, like *LncRNA HOTAIR*, *LncRNA ROR*, and *miRNA-14* have been linked to the onset and progression of OP (161, 162), and the expression levels of these ncRNAs have been discovered to be modulated by various active components in TCM, such as ginsenoside, curcumin and baicalin (163). Therefore, ncRNAs may serve as promising molecular targets for clinical therapeutic options of OP with TCM.

Some clinical trials focused on TCM in the treatment of OP have been carried out (82, 164–167), however, most of the clinical studies only contain a small sample size and short treatment duration, as well as the clinical parameters and biomarkers for analysis differ from each other, which results in a limited amount of robust evidence from clinical trials regarding effective approaches for preventing and treating OP. Thus, a sequential combination of TCM and Western medicine is the only way to solve these problems. In fact, some clinical trials on the treatment of OP by the combination of TCM and Western medicine have been carried out, such as Dihuang Decoction plus alendronate (83), Bushen Zhuanggu tablet plus calcium supplement and vitamin D (84), EXD plus caltrate tablets and calcitriol (102). A diverse array of sources from TCM formulas and their chemically active ingredients, along with ample anti-OP mechanisms, further enhance our assurance, eagerness, and motivation to discover new anti-OP drugs, but screening is imminent.

## Author contributions

YZ, CW, LZ, and HR: conceptualization. CZ, SS, and MZ: writing original draft preparation. All authors contributed to the article and approved the submitted version.

## Funding

This work was financially supported by National Natural Science Foundation of China (Nos. 82174140, 82174401, 81973870), Natural Science Foundation of Zhejiang Province (Nos. LY22H270003 and LQ19H080001), the Joint Funds of the Zhejiang Provincial Natural Science Foundation of China under Grant No. LBY22H270008, Traditional Chinese Medical Administration of Zhejiang Province (Nos. 2022ZX005, 2022ZB119, 2021ZB090), Zhejiang Medical and Health Science and Technology Project (Nos. 2023RC194, 2021KY222), Scientific Research Project of Zhejiang Chinese Medical University (Nos. 2021JKZDZC02, 2021JKZKTS036A, 2021JKJNTZ022B, 2019ZG25), Research Project of Zhejiang Chinese Medical University Affiliated Hospital (Nos. 2022FSYYZZ05 and 2022FSYYZQ02), Zhejiang Chinese Medical University School-level Education and Teaching Reform Project (Nos. CY22001), National Undergraduate Innovation and Entrepreneurship Training Program (Nos. 202210344064, 202210344006, 202210344028, 202210344037).

## Conflict of interest

The authors declare that the research was conducted in the absence of any commercial or financial relationships that could be construed as a potential conflict of interest.

## Publisher’s note

All claims expressed in this article are solely those of the authors and do not necessarily represent those of their affiliated organizations, or those of the publisher, the editors and the reviewers. Any product that may be evaluated in this article, or claim that may be made by its manufacturer, is not guaranteed or endorsed by the publisher.

## Figures and Tables

**Table:** 

OP	Osteoporosis
TCM	Traditional Chinese Medicine
BMD	Bone mineral density
BSHXD	Bushen Huoxue decoction
EXD	Er Xian Decoction
LWDHP	Liuwei Dihuang Pill
QEP	Qing’e Pill
DGBXD	Danggui Buxue Decoction
JQBD	Jianpi Qingchang Bushen decoction
BSTLD	Bushen Tongluo decoction
YGY	You Gui Yin
DHJSD	Duhuo Jisheng Decoction
THSWD	Taohong Siwu Decoction
ZGP	Zuogui Pill
OBs	Osteoblasts
OCs	Osteoclasts
BMSCs	Bone marrow mesenchymal stem cells
OVX	Ovariectomize
CLCF1	Cardiotrophin-like cytokine factor 1
GIOP	Glucocorticoid-induced osteoporosis
